# Effects of Exercise Programs on Physical Factors and Safety in Adult Patients with Cancer and Haematopoietic Stem Cell Transplantation: A Systematic Review

**DOI:** 10.3390/ijerph19031288

**Published:** 2022-01-24

**Authors:** Erica Morales-Rodriguez, Txomin Pérez-Bilbao, Alejandro F. San Juan, Jorge Lorenzo Calvo

**Affiliations:** 1 Sports Department, Faculty of Physical Activity and Sports Sciences-INEF, Universidad Politécnica de Madrid, 28040 Madrid, Spain; em.morales@alumnos.upm.es (E.M.-R.); jorge.lorenzo@upm.es (J.L.C.); 2 Department of Health and Human Performance, Faculty of Physical Activity and Sports Sciences-INEF, Universidad Politécnica de Madrid, 28040 Madrid, Spain; tperez@comillas.edu; 3 Department of Education, Investigation Methods and Evaluation, Faculty of Human and Social Sciences, Universidad Pontificia de Comillas, 28049 Madrid, Spain

**Keywords:** cancer, hematopoietic stem cell transplantation, bone marrow transplant, exercise, fitness

## Abstract

This study looks at the effects of exercise programs on physical factors and safety in adult patients with cancer and hematopoietic stem cell transplantation (HSCT) or bone marrow transplantation (BMT). A systematic search was performed in the PubMed and Web of Science databases (from inception to 26 August 2021). A review was carried out following the Preferred Reporting Items for Systematic review and Meta-Analyses (PRISMA) checklist. The methodological quality of the included studies was assessed with the Physiotherapy Evidence Database (PEDro) scale, based, in turn, on the Delphi list. A total of 25 randomized controlled trials studies were included, comprising 1434 patients. The most significant result of this systematic review is that exercise program interventions are safe and produce positive changes in cardiorespiratory fitness, muscle strength, and the functional mobility-state in adult patients with cancer and HSCT or BMT. Only 3 patients from the 711 participants in the exercise interventions (i.e., 0.42%) reported adverse events related to exercise interventions. Moreover, exercise training programs may have a cardiological and muscular protective effect, as well as a healthy effect on the prevention and control of transplant complications, improving health outcomes.

## 1. Introduction

Bone marrow transplantation (BMT), or hematopoietic stem cell transplantation (HSCT), is a treatment for hematological pathologies [1] such as myelosuppression produced by high dose chemotherapy (HDC), neoplastic syndromes, immunodeficiency, and enzyme deficiencies [2]. Autologous HSCT (i.e., stem cells come from the patient’s own blood or bone marrow) is the most common; it depends on the conditioning therapy that precedes transplantation and on hematopoietic growth factors that can reduce cytopenia after transplantation [3]. Allogeneic HSCT (Allo-HSCT), involving genetically similar donors, is associated with the side effects of treatment or graft versus host disease (GVHD) in 50% of its recipients [4]. The 5-year survival rate is 80% [5,6], with a 59% cumulative incidence of developing a disease [7].

Antineoplastic treatments have side effects [3] such as: the loss of physical performance [8], fatigue [9,10], and a weak immune and hematological system that sometimes lead to HSCT [11]. HSCT is related to the appearance of: pain, dizziness, asthenia, anemia, cytopenia, nausea, vomiting, diarrhea, infections, and fever related to aplasia and length of hospitalization [12]. Functional capacity is directly related to physical capacity as an indicator of health in pathological processes [13], and they serve for medical diagnosis [14]. Overall, 25% of cancer patients suffer a functional decline due to a decrease in physical activity, such as: loss of cardiovascular function, muscle strength, and lung function, as well as a reduction in lean body tissue [15].

In the past, physicians recommended patients with chronic diseases (e.g., cardiovascular diseases, pulmonary disorders, cancer) to avoid physical activity. However, in 1960s, numerous clinical studies showed the benefits of exercise on cardiac rehabilitation. These studies, together with the recommendations of the World Health Organization (1964), pushed the establishment and development of cardiac rehabilitation based on exercise [16]. In oncologic patients, the first report about the benefits of exercise was in 1980s, showing mood-enhancing effects during antineoplastic treatment [17]. In 1989, the first randomized controlled trial in 45 women with breast cancer under chemotherapy was realized. After 10 weeks of an aerobic exercise intervention, they observed a significant improvement in the maximum oxygen consumption (VO_2max_) [18]. From then on, the scientific evidence of exercise programs increased exponentially and pushed the establishment of oncologic rehabilitation based on exercise [19,20].

Specifically, the first study focusing on the functional capacity recovery of cancer patients after BMT was carried out by Dimeo et al. [21]. They developed an aerobic exercise program for 6 weeks and observed significant improvements in maximum physical performance and walking distance and a diminished heart rate with equivalent workloads [21]. It is currently known that exercise programs in patients with HSCT-BMT produce improvements in cardiorespiratory fitness (CRF), which is a predictor of mortality and cardiovascular diseases [22], the main indicator of which is the maximum oxygen consumption (VO_2max_). They also enhance muscle strength [1,23,24], metabolic and immune function [25], reduce the side effects of cancer treatment [26], and influence creatine, haemoglobin concentration, and hospitalization time [27,28]. However, there are limitations with existing studies, such as the joint inclusion of allogeneic and autologous patients [21,29], few control groups [30], or a small sample size [31].

The aim of this systematic review is to provide an updated analysis of data from experimental studies that have examined the effects of exercise programs on physical factors and safety in adult patients with cancer and HSCT or BMT.

## 2. Materials and Methods

### 2.1. Search Strategy

We used the Preferred Reporting Items for Systematic Reviews and Meta-analysis (PRISMA) to conduct this systematic review, as well as its possible consequences for the risk of bias [32]. An electronic search was realized for articles written in English in the electronic databases PubMed and Web of Science (from inception until 26 August 2021). We used the Mesh-indexed terms from PubMed and the following search strategy: (bone marrow transplant OR hematopoietic stem cell transplantation) AND (exercise OR physical activity) AND (immune system OR cardiovascular function OR neuromuscular function OR oxygen consumption OR strength OR resistance training OR acceleration OR mobility OR range of motion OR health OR neoplasms OR cancer survivors).

### 2.2. Selection of the Studies

Published pilot studies were included. Unpublished clinical trials registered in clinicaltrials.gov that provided results, grey literature (e.g., abstracts, conference proceedings, and editorials), and reviews were excluded. A difference was made between exercise programs pre- and post-HSCT in cancer patients. All the selected studies met the following inclusion criteria: (a) published in English; (b) randomized controlled trials (RCTs); (c) adult patients (age ≥18 years old) of both sexes who suffered or had suffered from any type of cancer at the time of the study; (d) patients in the process of receiving or who had received an HSCT; and (e) patients who had undergone an exercise program intervention. The studies exclusion criteria were: (a) participants under 18 years of age; (b) participants were not humans; (c) patients not related to cancer; (d) studies that did not include an exercise program; (e) studies related with other diseases or themes. The selection of the studies was realized by two authors independently (E.M.R and J.L.C.), and disagreements were resolved through discussion with a third author (T.P.B.).

### 2.3. Data Extraction

Two authors (E.M.R. and J.L.C.) independently extracted the following data from each study: sample (N); sex and age of participants; type of cancer; characteristics of the interventions (type, equipment, frequency, intensity, duration, session, rest, supervision, adjustment); relevant clinical variables and significant results. If there were disagreements in data extraction, authors discussed until a consensus was reached.

### 2.4. Risk of Bias Assessment

Two authors (E.M.R and J.L.C.) independently scored the studies, applying the Physiotherapy Evidence Database (PEDro) scale, based, in turn, on the Delphi list [33], and disagreements were resolved through discussion with a third author (T.P.B.). The total score of the PEDro scale was from 0 to 10, counting the number of criteria met by each study (see footnotes in Table 1). The quality of the study was rated as poor (PEDro score ≤ 3), fair (4–5), or high (≥6).

## 3. Results

### 3.1. Study Selection

**Figure 1:**
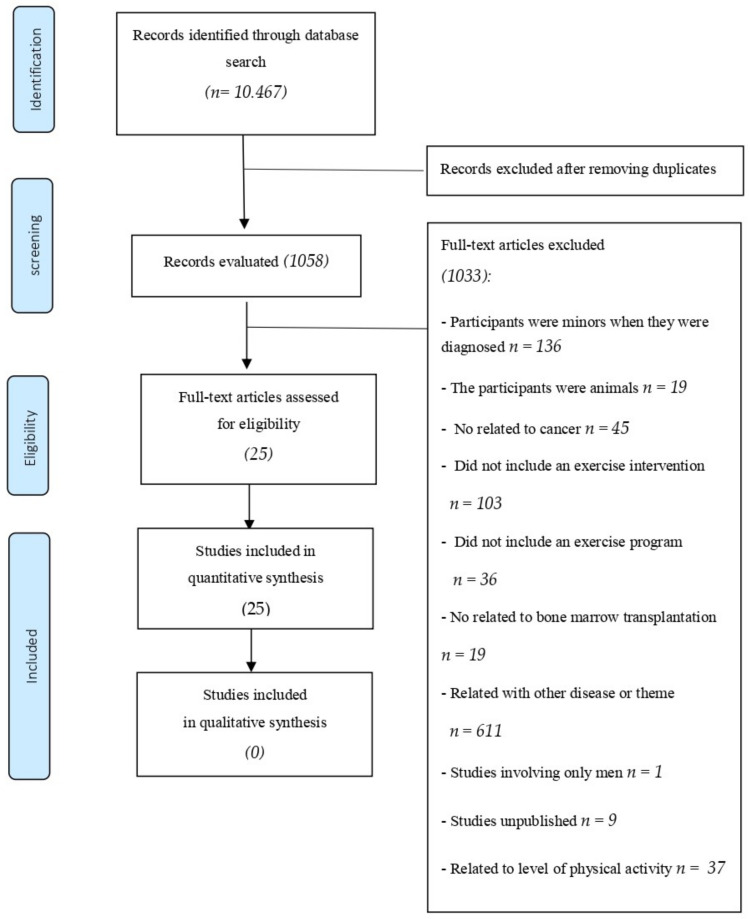
Systematic review flow chart.

A total of 10,467 references were identified in the database. After eliminating duplicate studies (*n* = 9409), 1058 studies remained. After selection by title, abstract, and full text, 1033 articles were excluded, and 25 studies met the inclusion criteria (Figure 1).

### 3.2. Study Characteristics

Twenty-five studies were included and analyzed in this systematic review [23,30,31,34,35,36,37,38,39,40,41,42,43,44,45,46,47,48,49,50,51,52,53,54,55] (Table 1). The characteristics of the included studies are summarized in Table 2 and Table 3. All studies included were RCTs with a control group (CT), with no exercise intervention, and an intervention group (EXP) except the study [34], which divided the sample size into a supervised or self-directed exercise program.

### 3.3. Quality Assessment and Publication Bias

The quality of the 25 included studies was high (median PEDro score = 6, range 6–10; Table 1). There were 4 articles with a score = 6, 17 articles with a score = 7–8, and 4 articles with a score = 9–10.

### 3.4. Characteristics of Participants

The analyzed studies included a total of 1434 oncologic patients (age range 18 to 75 years). They were distributed into an EXP with 711 patients (274 women) or a CT with 723 patients (250 women), although there were studies [37,39] that did not reflect the exact number of women in the experimental or control group so, there, more women could be involved. The most common cancers in the participants were leukemia, lymphoma, and myeloma.

### 3.5. Characteristics of Exercise Interventions

The characteristics of the exercise interventions (Table 2 and Table 3) were very diverse.

In total, 15 studies [23,30,31,36,38,39,40,42,45,46,47,48,49,54,55] analysed the effects of an exercise program intervention in cancer patients before and after HSCT treatment, and 10 studies analysed the effects after HSCT [34,35,37,41,43,44,50,51,52,53].

*Supervision*: Exercise programs were supervised by researchers in 21 studies [23,31,34,35,37,38,40,41,42,43,44,45,46,47,49,50,51,52,53,54,55] and semi-supervised in 2 studies [36,48] and 2 studies was not supervised [30,36].

*Frequency*: A total of 13 studies (52%) performed exercise intervention 5 times per week [23,31,36,38,40,42,44,45,46,47,52,54,55]; 5 studies (20%) performed intervention 3 times per week [34,37,48,49,51]; 3 studies (12%) increased the exercise frequency to 7 times per week [30,35,41]; and 3 studies (12%) exercised twice per week [43,50,53]. Only 1 study (4%) did not report exercise frequency [39].

*Intensity training:* Six studies (24%) did not adequately report intensity exercise [30,39,41,44,45,54], two studies (8%) offered no information regarding strength training [34,43] and one study (4%) did not report information regarding aerobic training [52]. In 10 studies (40%), the intensity exercise was evaluated using a perceived exertion scale (RPE) [31,36,37,39,40,42,47,48,49,51], 7 studies (32%) reported aerobic intensity exercise using a percentage of heart rate maximal (HRmax) [23,31,34,40,42,43,46], 3 studies (12%) reported intensity using a percentage of 1 repetition maximum (1RM) [50,52,53], 3 studies (12%) reported aerobic intensity exercise using a percentage of power [38,50,53], and 2 studies (8%) reported the intensity exercise using a percentage of maximal inspiratory pressure (MIP) [35,55].

*Duration:* A total of 14 studies (56%) lasted up to 8 weeks [23,31,34,35,37,38,40,41,42,45,46,48,49,52], 7 studies (28%) lasted between 9 and 18 weeks [30,36,43,47,50,51,53], and 1 study (4%) lasted more than 18 weeks [39]. This information was not fully reported in three studies (12%) [44,54,55]. The most common duration was six weeks, with eight studies (32%) being performed for this duration. The duration per session ranged from 15 to 60 min [30,31,40,42,53,54,55], and the majority of the studies included interventions that lasted either between 20 to 40 min (seven studies (28%) [23,35,38,41,44,45,47]) or 60 min (four studies (16%) [31,40,42,53]). Overall, 11 studies did not adequately report the duration of the exercise sessions [34,36,37,39,43,49,50,51,52,54,55].

*Type*: The majority of the studies included multicomponent exercise interventions. Nine studies (36%) focused on strength and aerobic training [34,36,39,43,45,47,50,52,53], three studies (12%) included strength, aerobic training, stretching and relaxation [31,40,42], two studies (8%) included aerobic and activities of daily living (ADL) training [23,38], one study (4%) included stretching, aerobic and mobilization training [46], one study (4%) included inspiratory muscle training (IMT) [35], one study (4%) included stretching, mobilization and relaxation [41], one study (4%) included aerobic training [30], one study (4%) included strength and mobility training [48], one study (4%) included strength training [37], two studies (8%) included strength training plus whole body vibration (WBV) [49,54], one study (4%) included aerobic training and relaxation [51], one study (4%) included aerobic training, strength and stretching [44], and one study included IMT, aerobic training, ADL, stretching, coordination and balance [55]. When reported, the most-used activities for aerobic training were walking and cycling. For strength training, whole-body exercises were used with or without equipment, although the most common was the use of elastic bands. In the case of respiratory muscle exercises, pressure threshold-loading devices were used. For strength training plus whole-body vibration, a vibration platform was used. For a more detailed description, please see Table 2 and Table 3.

*Delivery setting:* Fourteen interventions (56%) were delivered in health care settings (e.g., hospitals) [23,31,38,40,41,42,44,46,49,50,52,53,54,55], nine interventions (36%) were delivered both in the participant’s homes and in health care settings [30,34,35,36,37,45,47,48,51], one intervention (4%) was delivered in the participant’s home [39], and one intervention (4%) was delivered both in the physiotherapy practice and in the fitness centre [43].

### 3.6. Exercise Safety—Related Adverse Events

A total of 7 of the 25 studies (28%) reported adverse effects. One study (4%) reported a calf muscle strain during a non-exercise related training session [50], and two studies (8%) reported exercise-related adverse effects [39,55], while four studies (16%) reported adverse effects, but it was unclear whether they were due to the exercise intervention [23,30,36,40].

The exercise programs seem to be safe in most of the interventions included in the present review (i.e., 23 studies), and only 2 studies from the 25 were clearly associated with adverse events [39,55]. Furthermore, only 0.42% of the patients (3 from the 711 participants in the exercise interventions) suffered these adverse events: central venous catheter rupture in an exercise test (1 patient; 0.14%) [39], vomiting (1 patient; 0.14%), and desaturation (1 patient; 0.14%) [55].

### 3.7. Compliance Rate

A total of 12 out of 25 papers (48%) reported the rate of adherence of their participants to exercise, with the compliance rate of the participants being: *<40%* [30,34], *40–75%* [37,39,54], *80–90%* [40,48,50], *>90%* [31,36,55], and *not specified* [49] (see Table 2 and Table 3 for a more detailed description of the studies).

### 3.8. Endpoints and Exercise Intervention Results

#### 3.8.1. Cardiorespiratory Fitness (CRF)

CRF was analysed in 17 of the 25 studies (68%). The following lung function variables were measured directly with a gas analyser, spirometer, and flow screen: VO_2max_ [31,42,50,52,53,54], ventilation carbon dioxide (VCO_2_) [52], expiratory minute ventilation volume (VE) [52], inspiratory vital capacity (IVC) [23], vital capacity (VC) [38], forced vital capacity (FVC) [23,35,38], MIP [35,55], maximal expiratory pressure (MEP) [35], forced expiratory volume in the first second/forced vital capacity (FEV1/FVC) [35], modified medical research council (MMRC) [35], forced expiratory volume in the first second (FEV1) [35], peak expiratory flow (PEF) [35], and forced expiratory flow from 25% to 75% (FEF 25–75%) [35]. Other variables measures in CRF were: 2-min stair climb test (2MWT) [31,42,44], 6-min walk test (6MWT) [34,35,36,43,45], shuttle walk test (SWT) [51], and walk on treadmill (min/watts) [39,44]. In addition, submaximal aerobic endurance [38] and relative endurance [23,38], with a submaximal endurance test based on the World Health Organization recommendations (WHO), were measured.

In 11 out of 17 articles (65%), the results indicate a significant improvement in the following variables in CRF: VO_2max_ [42,52,54], MIP [35,55] and MEP [35], MMRC [35], 6MWT [34,35,36,43,45], and relative endurance [23,38].

#### 3.8.2. Muscle Strength and Power

Muscle strength was analyzed in 18 of the 25 studies (72%). Skeletal muscle strength was determined in 16 of the 25 studies (64%): Maximal isometric voluntary strength tests (MIVS) were obtained from muscle groups of the upper limbs [52], hand-grip strength (HGS) [35,37,43,44,45,50,53], abdominal muscles [50,52,53], exercises of the autochthon back and arm muscles [52], only lower extremities [54], upper and lower limbs [35,37,42,43,44,45,50,53]. Muscle power was analyzed in 2 of the 25 studies (8%), with the following variables: jumping height [49,54] and maximum power output (Pmax) [54].

In 7 of the 16 papers of skeletal muscle strength (44%), the results indicate an improvement in the following variables: upper limb muscle strength [39,46,52], right elbow flexor [42], elbow extension [45], chest press [42], hip flexion [45], right knee flexion [31], knee extension [42,43], leg extension [31,42], lower extremities [39,46] and Strength Training to Enhance Early Recovery (STEER) [48]. Moreover, in both studies of muscle power (100%), the results indicate that an improvement was observed in terms of an enhancement of the jumping height [49] and Pmax in a counter-movement jump test [54].

#### 3.8.3. Functional Mobility State

Functional mobility and functional state was analyzed in 8 of the 25 studies (32%) using the following variables: modified incremental shuttle walk test (MISWT) [35], 50-foot walk test (50 FWT) [34,43], 30 s chair stand test (30 CST) [34,37,50,54], timed-up-and-go test (TUG) [49], time needed to stand up (TNSU) [37], semi-tandem stance with eyes open (ST_EO_) and with eyes closed (ST_EC_) [49], step activity monitor (SAM) [43], physical activity scale for the elderly (PASE) [50], forward reach test [34], and Karnofsky performance status (KPS) [30].

In 6 of the 8 studies (75%), the results indicate an improvement in the following functional state variables: MISWT [35], 50 FWT [43], 30 CST [37,54], TUG [37,49], ST_EO_ and ST_EC_ sway path [49], and KPS [30].

#### 3.8.4. Body Composition

Body composition was analysed in 18 of the 25 studies (72%) using body mass index (BMI) [23,31,35,36,38,40,42,43,45,46,47,49,50,52,54,55], lean body weight (LBW) [38,39,43,49], weight [30,35,38,43,52], body height [35,38,52], fat mass [43], body fat [54], fat free mass (FFM) [43,54], body cell mass [54], and phase angle [54]. In 1 of the 18 studies (6%), the exercise program produced a significant improvement in LBW [39].

Some of the techniques mentioned to measure body composition were the following: LBW with air-displacement plethysmography [36,39,51], fat mass via dual-energy X-ray absorptiometry (DXA) [43], and body cell mass and phase angle with bioelectrical impedance analysis (BIA) [43,54]. All the techniques agreed that they were performed before and after transplantation.

### 3.9. Immune System

The immune system was analyzed in 3 of the 25 studies (12%) using the following variables: leukocytes [23,42], lymphocyte count [41], platelets [23,42], and hemoglobin [23]. None of the three articles showed that the exercise program produced a significant improvement or deterioration of blood parameters. Only one study maintained normal values of hematological lymphocytes (1000–4500 cells/µL) [41]. Further, the characteristics (i.e., type of cancer and treatment) of the studies analyzed are as follows: two of the three studies (67%) used the Allo-HSCT treatment with conditioning therapy (i.e., a combination of chemotherapy, radiotherapy and/or immunotherapy) with total body irradiation (TBI) in patients with AML, ALL, and CML [23,42]. Lastly, one of the three studies (33%) used the HSCT-BMT treatment combined with conditioning therapy in patients with AML, and ALL [41]

## 4. Discussion

This study reviews the scientific literature on the effects of exercise programs on physical factors and safety in adult patients with cancer and HSCT-BMT. The most significant result of this systematic review is that training program interventions seem to be safe in adult patients with cancer and HSCT and produce significant improvements in CRF, muscle strength and power, functional mobility, and functional state. To our knowledge, there are two systematic reviews and meta-analyses, published in 2013 [56,57], which studied the effects of exercise on health factors, and both included nine studies that are also analyzed in our review. Our systematic review presents similarities with these two reviews [56,57]: (a) only RCTs published in English were included; (b) exercise interventions varied widely; (c) exercise interventions were safe and well tolerated; (d) exercise programs improved physical factors. On the other hand, our review presents some differences from these previous reviews [56,57]: (a) we did not perform a meta-analysis; (b) our review included a larger number of RCTs (25 vs. 11 and 8 studies, respectively).

### 4.1. Cardiorespiratory Fitness

Of the 25 papers, in 11 of 17 articles (65%), improvements were observed in the measured variables of CRF. Our findings on CRF show that exercise interventions increased the performance, on average, by 6.6% (3.1–11.3%) in the VO_2max_, 9.2% (0.2–14.5%) in the 6MWT, and 13.9% (11.0–16.7%) in relative endurance. In the case of the 6MWT, there was a study without improvement, but it showed maintenance of the baseline values (−0.19%) compared to a decrease in the control group (−9.9%) [36]. The 6MWT is a predictor of morbidity and mortality as well as a measure of the patient’s functional status [58], and VO_2max_ is the most important variable related to CRF [28]. Therefore, exercise seems to have a healthy effect on the heart, prior to and after transplantation, maintaining or even increasing the physical capacity of the individual. The exercise intervention also prevents a loss of physical performance, according to Dimeo et al. [21].

On the other hand, 6 of the 17 articles (35%) did not observe significant improvements after the exercise interventions and, although VO_2max_ was maintained in the intervention group, it was not statistically significant [31,50,53]. The lack of improvements may be due to the following issues: (a) the intervention group received self-training on weekends (mean 4.5 days) during hospitalization [31]; (b) the training frequency of the physical activity program was low (i.e., twice a week over 18 weeks) [50,53]; (c) there was a lack of control and rigorous planning of the variables of the exercise program (e.g., intensity, frequency, volume) and poor compliance [50]; (d) a large number of VO_2peak_ values were lost (36%), and only 23% of the patients examined participated in the study [50]; (e) VO_2max_ was measured in patients after HSCT [53], and it is possible that this measurement does not reflect an improvement in the patient’s health status, as a post-transplant exercise program does not necessarily accelerate the patient’s recovery process [31,53]. However, there are studies that show that VO_2max_ not only decreases due to treatment but can also be reduced due to bed rest during the first 10 days of inactivity [59].

### 4.2. Muscle Strength and Power

Of the 25 papers, in 10 of 16 studies (62.5%), a mean increment of 11.2% (3.5–26.2%) is observed in the measured variables of skeletal muscle strength. Moreover, some studies [31,39,46] showed the maintenance of the initial strength values in the groups of exercise intervention, with a mean improvement of 1.4% (0.1–4.1%). However, the control groups in these studies showed a significant loss in their initial muscle strength capacity after the HSCT, showing a mean decrease of 5.4% (−0.4–9.1%). Then, the exercise program plays an important protective role over the muscle capacities during the most aggressive treatment phases. On the other hand, in two out of two studies (100%), improvements are observed in the measured variables of power, with an average increase of 18.6% (12.4–24.8%). Strength training improves muscle mass and reduces its catabolic wear from cancer-related treatments [60] and counteracts the weakened strength observed after HSCT [61]. For these reasons, it is clinically relevant to reinforce physical functioning before and after HSCT to prevent secondary health problems [62,63]. Medical treatment alone has been extensively researched and provides fewer benefits than its implementation with an exercise program [64].

In contrast, in 6 of the 16 articles (37.5%), no significant improvements in skeletal strength were observed after the exercise intervention, which may be due to the following issues in different studies: (a) a small sample size [35,37,44,54]; (b) there was a gender imbalance between semi-supervised groups [35,37,44,50,53]; (c) other studies did not detail some of the units of measurements on the lower limb [37,53]; (d) in the study of Persoon et al. [50], despite having a good methodological design, no improvements were reflected due to non-progressive training planning (i.e., increase of volume and intensity) and low compliance; (e) in Van Dongen’s intervention, in the last 12–18 weeks, the training frequency was reduced to once a week [53]; (f) in another study with a 6-week follow-up, in which the control group was given exercise recommendations and guidelines, 4 weeks after discharge, two patients in this group exercised, and this may be a confounding factor [37]; and (g) the IMT specific for respiratory muscles was not oriented towards gaining limb strength [35]. In another paper, no comparison was made between the WBV training and conventional resistance training during transplantation; therefore, the reported results could not be explicitly attributed to WBV training being more beneficial than conventional training [54].

### 4.3. Functional Mobility and Functional State

Of the 25 papers, in six of eight articles (75%), a mean improvement of 13.6% (5.5–19.4%) was observed in the measured variables of functional mobility and functional state. In the specific case of the KPS [30], the intervention group worsened less than the control group, with a decrease of 10 points compared to the 20 points lost by the control group (KPS scale ranges from 0 to 100 points). However, in two of the eight articles [50] (25%), no significant improvements are observed after the exercise interventions, which may be due to the following issues: (a) the time of the intervention was just after BMT [50], and there are studies that argue that exercise does not speed recovery immediately after HSCT [43]; (b) the control group was not restricted in physical activity or access to physiotherapy services, and this may have altered the results [50]; (c) the level of adherence (23%) was very low [50]; (d) a low recruitment rate [34]; (e) the variation in the volume of physical activity between both groups [34]; (f) the lack of daily quantification of physical activity levels [34]; and (g) a program duration of only 4 weeks [34].

### 4.4. Body Composition

Of the 25 papers, in 1 of 18 articles (6%) [39], a 0.1% increment in LBW was observed. It should be noted that in this case, the small increase means that the exercise intervention recovered the baseline values while the control group presented LBW losses of 3.6%. However, in 17 of the 18 articles (94%), no significant improvements were observed after the exercise intervention, which may be due to the following issues: (a) the time of exercise intervention in relation to treatment, as it was applied to patients undergoing intensive chemotherapy during hospitalization, with a really high catabolic environment [49,50]; (b) a small sample size [40,49,54]; (c) difficulty in the monitoring of patients due to timetables and transport restrictions [40]; (d) the intervention was semi-supervised [40]; (e) within the sample size, the distribution of patients between both groups was not homogeneous, with a minority number of women in each group: EXP (N = 64; 26 female), TC (N = 67; 28 female) [43]; (f) low intensity, frequency and duration of the program in relation to the guidelines of the American College of Sports Medicine (ACSM) [43]; and (g) a drop-out rate greater than 20% [43].

## 5. Limitations

This systematic review demonstrates that the research published so far on this topic has limitations with respect to: (a) the small sample size of the groups (17 of 25 studies including <100 participants); however, it must take into account that sometimes it may be hard to collect data from numerous patients with the same cancer type and treatment, and to meet all the inclusion and exclusion criteria; (b) the shortage of control groups [31,37,39,46,49]; (c) the heterogeneous characteristics of the sample (e.g., wide age range, different types of cancer, and time elapsed since diagnosis and from the end of treatment); (d) the heterogeneity of exercise interventions (e.g., different types, frequency, intensity, type of session, volume of session, type of supervision); (e) the lack or short duration of the follow-up [34,49,52]; and (f) the control of exercise compliance rate accomplished in only 12 out of 25 items [30,31,34,36,37,39,42,48,49,50,54,55];. There is low scientific evidence about the effects of exercise on the immune system of these patients, although it appears that exercise interventions have maintained normal values. However, they cannot be generalized, and it will depend on the type of cancer and the treatment administered [23,36,41,42]. Future lines of research should focus on RCTs to advance towards new exercise programs with different load planning, larger sample size, greater homogeneity of the sample, detailed control of the effect of exercise on the immune system, and long-term follow-up of the exercise program.

## 6. Conclusions

In conclusion, it seems that exercise program interventions are safe and produce benefits on CRF, muscle strength and power, and functional mobility and states in adult patients with cancer and HSCT. Thus, exercise training programs may have a cardiological and muscular protective effect as well as a healthy effect on the prevention and control of transplant complications, improving health outcomes. However, more scientific evidence, such as RCTs or meta-analyses, is still necessary to confirm these findings and improve the highest quality evidence.

Our results show that exercise program interventions are clinically relevant, and it is necessary to implement them in a generalized way in these patients. Most of the programs analyzed were supervised, multicomponent (i.e., strength, aerobic, stretching, mobilization, and activities of daily living training), 3 to 7 days a week, with a duration from 6 to 18 weeks, with a session duration of 20 to 60 min, and developed at health care settings or at the patient’s home (depending on the patient status). Further, to improve the health and quality of life benefits produced by the program, the exercise load and intensity must be individualized and controlled during the training process.

More research is needed regarding exercise tolerance in severely immunosuppressed patients, as well as research on improving body composition through concurrent exercise and nutrition interventions.

## Figures and Tables

**Table 1:** Quality of the studies included in the systematic review.

Items												
Study	1	2	3	4	5	6	7	8	9	10	11	Total Score
(Baumann et al., 2010) [23]	+	+	+	+	-	-	-	+	+	+	+	7
(Jarden et al., 2007) [31]	+	+	+	+	+	?	?	+	+	+	+	9
(Shelton et al., 2009) [34]	+	+	+	+	-	-	-	+	+	+	+	7
(DeFor et al., 2007) [30]	+	+	+	?	?	?	?	+	+	+	+	6
(Barğı et al., 2016) [35]	+	+	+	+	+	+	-	+	+	+	+	9
(Wiskemann et al., 2011) [36]	+	+	+	+	?	?	?	+	+	+	+	7
(Hacker et al., 2011) [37]	+	+	+	+	?	?	?	+	+	+	+	7
(Baumann et al., 2011) [38]	+	+	+	+	-	-	-	+	+	+	+	7
(Coleman et al., 2003) [39]	+	+	+	+	?	?	?	+	+	+	+	7
(Jarden et al., 2009) [40]	+	+	+	+	+	-	-	+	+	+	+	8
(KIM & KIM, 2006) [41]	+	+	+	+	-	-	-	+	+	+	+	7
(Jarden et al., 2009) [42]	+	+	+	+	-	-	-	+	+	+	+	7
(Knols et al., 2011) [43]	+	+	+	+	+	-	+	+	+	+	+	10
(Schumacher et al., 2018) [44]	+	+	+	+	+	?	?	+	+	+	+	8
(Wiskemann et al., 2014) [45]	+	+	+	+	+	-	-	+	+	+	+	8
(Mello et al., 2003) [46]	+	+	?	+	?	?	?	+	+	+	+	6
(Wiskemann et al., 2015) [47]	+	+	?	+	?	?	?	+	+	+	+	6
(Peters et al., 2018) [48]	+	+	+	+	+	-	-	+	+	+	+	8
(Pahl et al., 2018) [49]	+	+	+	+	-	-	-	+	+	+	+	7
(Persoon et al., 2017) [50]	+	+	+	+	+	+	+	+	+	+	+	10
(Bird et al., 2010) [51]	+	+	+	+	-	-	-	+	+	+	+	7
(Oechsle et al., 2014) [52]	+	+	-	+	-	-	-	+	+	+	+	6
(Van Dongen et al., 2019) [53]	+	+	+	+	-	-	-	+	+	+	+	7
(Pahl et al., 2020) [54]	+	+	+	+	+	-	-	+	+	+	+	8
(Almeida et al., 2020) [55]	+	+	+	+	+	-	-	+	+	+	+	8

Column numbers correspond to the following criteria on the PEDro scale: 1—Eligibility criteria were specified. 2—Subjects were randomly allocated to groups (or, in a crossover study, subjects were randomly allocated an order in which treatments were received). 3—Allocation was concealed. 4—Groups were similar at baseline. 5—Subjects were blinded. 6—Therapists who administered the treatment were blinded. 7—Assessors were blinded. 8—Measures of key outcomes were obtained from more than 85% of subjects. 9—Data were analyzed by intention to treat. 10—Statistical comparisons between groups were conducted. 11—Point measures and measures of variability were provided. A total score out of 10 is determined from the number of criteria that are satisfied, except that scale item 1 is not used to generate the total score. + Indicates the criterion was clearly satisfied; - indicates that it was not; ? indicates that it is not clear whether the criterion was satisfied.

**Table 2:** Studies that have analyzed the effects of an exercise program before and after HSCT, with main results in physically related variables.

Study	Study Design	Sample Size by Group (Sex), Age (Mean ± SD; Range)	Primary Cancer	Intervention	Main Results in Physical Related Variables
(DeFor et al., 2007) [30]	RCT T1: Pre HSCT (A) T2: Post HSCT (100 days)	- EXP: *n* = 51 (22 female), 46 years (18–68) - CT: *n* = 49 (17 female), 49 years (22–64)	AA, ALL, AML, CML, HL, LHN, MDS	Type: AT (walking on treadmill) Duration: 100 days Intensity: Comfortable speed Supervised: not Frequency: 7 times/week Setting: Clinic/home	T1-T2: - ↓ KPS: ↓ EXP (-10 pts/100 pts) ↓ CT (-20 pts/100 pts) - ↔ Immune system
(Wiskemann et al., 2011) [36]	RCT T1: Pre HSCT (medical checkup)-Pre HSCT (4 week A) T2: Post HSCT (H)-Post HSCT (D) T3: Post HSCT (D)-Post HSCT (D 6–8 weeks)	- EXP: *n* = 52 (21 female), 47.6 years (18–70) - CT: *n* = 53, (13 female), 50 years (20–71)	AA, ALL, AML, CLL, CML, MDS, MM, MPS, Others	Type: AT (walking, stationary bicycle), ST (elastic bands) Duration: 18 weeks Intensity and volume: AT 1–4 week to (A) 3 times a week, from (H) 3–5 times a week RPE (12–14/20), DCT: color codes (Red 15–20 min, yellow 20–30 min, green 30–40 min), from 1–8 week rehabilitation 3 times a week. ST 1–4 week to (A) twice a week, from (H) twice a week (2–3 sets of 8–20 reps RPE 14–16/20), from 1–8 week rehabilitation twice a week. Supervised: yes T2 and self-directed T1 and T3 Setting: Home/Hospital	T1 - ↑ 6MWT (∆-0.19%) - ↔ Immune system
(Baumann et al., 2011) [38]	RCT T1:Pre HSCT (A) T2:Post HSCT (7–8 weeks)	- EXP: *n* = 17, (6 female), 41.41 ± 11.78 ^a^ years - CT = *n* = 16, (11 female), 42.81 ± 14.04 ^a^ years	ALL, AML, CLL, CML, MDS, MM, MPS, PID	*Type:* AT (stationary bicycle), ADL, stretching, coordination *Duration:* 7–8 weeks *Volume and intensity:* AT (H) once-twice a week (10–20 min/day continuous or interval training at HRmax 80%); ADL-training (H) 5 times a day (20 min a day, 5 × 20 steps with 1 min break of slightly strenuous or strenuous); mobilization passive and active 1 day after HSCT until 1 day before hospital discharge daily except on weekends Low intensity; or not strenuous (CT) 20 min a day Cadence: AT cycle (since 25 W with 25 W increment every 2 min) *Supervised:* yes *Setting*: hospital	T1-T2: - Endurance: ↓ CT (∆-23.5%) - Relative endurance: ↓ CT/↑ EXP (∆-15.8%; ∆11.02%) - Strength lower extremities: ↓ CT (∆-26.8%) - BMI: ↓ CT/↓ EXP (∆-8.2%; ∆-13.2%) - ↔ Immune system
(Coleman et al., 2003) [39]	RCT with RM T1:Pre HSCT (A) T2: Post HSCT (3 months)	- EXP: *n* = 14 - CT: *n* = 10; (10 female), 55 years (42–74)	MM	*Type:* AT (walking), ST (elastic bands) *Duration:* 6 months *Intensity and volume:* AT 3 times a week (18 min fast-paced walking at RPE 12–15/20), ST 3 times a week with color bands (1 set of 8 red 9–15 Ib, 1 set of 8 green 5–9 Ib) and (2 sets of 8 chair stands of 1 RM) *Supervised:* not *Setting*: home	T1-T2: - LBW: ↑ EXP/↓ CT (∆0.1%; ∆-3.6%) - Strenght: ↑ EXP/↓ CT (∆2.4%; ∆-12.6%) - ↔ Immune system
(Jarden et al., 2009) [42]	RCT T1: Pre HSCT (A) T2: Post HSCT (6 weeks)	- EXP: *n* = 21, (8 female), 40.9 years (18–60) - CT: *n* = 21, (8 female), 37.4 years (18–55)	AA, ALL, AML, CML, MDS, MF, PNH, WM	*Type:* AT (stationary bicycle), ST (hand and ankle weights), stretching, relaxation *Duration:* 4–6 weeks Intensity and *volume:* AT 5 times a week (HRmax 50–75% Low to moderate RPE 10/13), stretching (Dynamic: 1–2 sets of 10–12 reps; Static: 1 set/15–30 seg), ST 3 times a week (1–2 sets of 10–12 reps at low to moderate, RPE 10/13) and relaxation twice a week (Low RPE 6/9) *Cadence:* 30–70 cycles/min and range at 30–75 W. *Supervised:* yes *Setting:* Hospital	T1-T2: - ↔ Immune system
(Jarden et al., 2009) [40]	RCT T1: Pre HSCT (A) T2: PostHSCT (D)	- EXP: *n* = 21 (8 female), 45.0 years (18–60) - CT: *n* = 21 (8 female), 38.0 years (18–55)	AA, ALL, AML, CML, MDS, MF, PNH, WM	*Type:* AT (stationary bicycle), ST (hand and ankle weights), stretching, relaxation *Duration:*4–6 weeks Intensity and volume: AT 5 times a week Low to moderate (HRmax 50–75% of RPE 10/13), stretching (Dynamic: 1–2 sets of 10–12 reps; Static: 1 set/15–30 sg), ST 3 times a week low to moderate, (1–2 sets of 10–12 reps at RPE 10/13) and relaxation twice a week (Low RPE 6/9) Cadence: 30–70 cycles/min and range of 30–75 W. *Volume:* ST, stretching and relaxation (Dynamic: (1–2 sets of 10–12 reps); Static: (1 set 15–30 sg) *Supervised:* yes Setting: Hospital	T1-T2: - VO_2_: ↑ EXP/↓ CT (∆3.1%; ∆-28.6%) - Chest press: ↑ EXP/↓ CT (∆5.3%; ∆-18.6%) - Leg extension: ↑ EXP/↓ CT (∆4.3%; ∆-30.3%) - Right elbow flexor: ↑ EXP/↓ CT (∆6.4%; ∆-23.1%) - Right knee extensor: ↑ EXP/↓ CT (∆3.5%; (∆-21.3%) - 2MWT: ↓ EXP/↓ CT (∆-26.0%; ∆-11.3%) - ↔ Immune system
(Wiskemann et al., 2014) [45]	Multicenter RCT T1: Pre HSCT (baseline)-Pre HSCT (A) T2: Pre HSCT (A)-Post HSCT (D) T3: Post HSCT (D)-Post HSCT (6–8 weeks)	- EXP: *n* = 52, (21 female), 47.6 years (18–70) - CT: *n* = 53, (13 female), 50 years (20–71)	AA, ALL, AML, CLL, CML, Lymphoma, MDS, MM	*Type:* AT (N/R), ST (elastic bands) *Duration:* 8 weeks. Frequency: AT: T1 (3 times a week), T2 (5 times a week), T3 (3 times a week). ST: T1, T2, and T3 (twice a week). Intensity and volume: Not specified *Supervised:* yes *Setting:* home/hospital	T1-T3: EXfit: - ↓ Knee extensión (∆-31.3%) - ↓ Hip flexion (∆-16.4%) - ↓ Elbow extensión (∆-21.2%) - ↓ Elbow flexion (∆-22.1%) - ↓ 6MWT (∆-4.3%) T1-T3: Exunfit: - ↓ Knee extensión (∆-8.6%) - ↑ Hip flexion (∆10.3%) - ↑ Elbow extensión (∆3.4%) - ↓ Elbow flexion - ↑ 6MWT (∆10.4%) - ↔ Immune system
(Baumann et al., 2010) [23]	RCT T1: Pre HSCT (A) T2: Post HSCT (D)	- EXP: *n* = 32, (11 female), 44.9 ± 12.4 ^b^ years - CT: *n* = 32, (18 female), 44.1 ± 14.2 ^b^ years	ALL, AML, CLL, CML, LHN, MDS, MM, Solid tumour, immuno-deficiency	*Type:* AT (stationary bicycle), ADL Duration: 7 weeks *Volume:* AT twice a week (80% HRmax), ADL 5 times a week (5 × 20 steps with 1 min break RPE ‘slightly strenuous’ to ‘strenuous’. CT 5 times a week *Cadence:* AT (increase 25 W/2 min) *Supervised*: yes *Setting:* hospital	T1-T2: - Relative endurance: ↑ EXP/↓ CT (∆16.7%; (∆-16.7%) - Plts: ↓ EXP/↓ CT - Hb: ↓ EXP/↓CT - Leucocytes: ↓ CT
(Jarden et al., 2007) [31]	RCT T1: Pre HSCT (A) T2:Post HSCT (±1 day D)	- EXP: *n* = 6 (2 female), 34 years (18–58) - CT: *n* = 8 (4 female) 37 years (18–53)	AA, ALL, AML, CML, hemoglobinuria, LHN, MF, MM, PNH	*Type:* AT (stationary bicycle), ST (hand and ankle weights), stretching, relaxation *Duration:* 6 weeks *Intensity and volume:* AT 5 times a week (Low to moderate 50–75% HR max and RPE 10–13/20), ST 3 times a week (Low to moderate 1–2 sets of 10–12 reps of 1RM at RPE 10–13/20), stretching 5 times a week (Dynamic: 1–2 sets of 10–12 reps Static: 1 set, hold for 15–30 s), relaxation 2 times a week (Low RPE 6–9/20), Psychoeducation 5 times a week *Cadence:* 30–70 cycles since 50 W *Supervised:* yes Setting: hospital	T1-T2: - Chest press:↓ CT (∆-14.9%) - Leg ext: ↑ EXP/↓ CT (∆6.1%; ∆-16.3%) - Right knee flex: ↑ EXP/↓ CT (∆0.2%; ∆-14.5%) - ↔ Immune system
(Mello et al., 2003) [46]	RCT T1: Pre HSCT (A)-Post HSCT (D) T2: Post HSCT (6 weeks)	- EXP: *n* = 9, (4 female), 27.9 years (18–39) - CT: *n* = 9, (6 female), 30.2 years (18–44)	AML, CML, LHN, MDS, SAA	Type:AT (walking), stretching, mobilization *Duration*: 6 weeks *Volume:* AT, range of motion and stretching (5 sets of 3 min comfortable walk to 2 sets of 10–20 min at 70% HR max speed walk in the sixth week) *Rest:* 3 min *Supervised*: yes *Setting*: hospital	T1-T2: - Knee DM-NDM Flexors: ↓ EXP/↓ CT - Shoulder:DM–NDM: ↓ CT - Elbow DM flexors/NDM: ↓ CT - Ankle DM/NDM Flexors: ↓ CT - ↔ Immune system
(Wiskemann et al., 2015) [47]	Multicenter RCT T1: Pre HSCT (A) T2: Post HSCT (D)	- EXP: *n* = 50, (21 female), 48.2 ± 14.5 ^b^ years - CT: *n* = 53, (13 female), 50 ± 12.4 ^b^ years	AA, ALL, AML, CLL, CML, Lymphoma, MDS, MM	*Type*: AT (walking, stationary bicycle), ST (elastic bands) *Duration:* 15–18 weeks *Volume and Intensity:* AT 3–5 times a week (RPE 12–14/20), ST twice a week (RPE 12–14/20) *Supervised*: yes *Setting*: home/hospital	T1-T2: - No changes
(Peters et al., 2018) [48]	RCT T1: Pre HSCT (H) T2: Post HSCT (D) T3: Post HSCT (6 weeks)	*n* = 70, 53.1 ± 13.5 years - EXP: *n* = 37 (15 female) - CT: *n* = 33 (13 female)	ALL, AML, CLL, CML, HL, LHN, MDS, MM	Type: ST (elastic bands), range of motion Duration: 6 weeks Intensity: ST T2-T3 (RPE 13–14/20) Intensity and volume: ST and range of motion: T2-T3 three times a week for 16 weeks with 18 sessions (1–2 sets of increase reps with bands) Supervised: not T1-T2 and yes T2-T3 Setting: hospital/home	- No changes
(Pahl et al., 2018) [49]	RCT pilot study T1: Pre HSCT (Admission) T2: Post HSCT (D)	- EXP: *n* = 6 (1 female), 47 years (19–62) - CT: *n* = 5 (2 female), 56 years (32–63)	ALL, AML, APL, HL, LHN, MM, MW, PMF	Type: AT stationary bicycle (CT), and ST with vibration (EXP) Duration: 27 days Intensity: AT and ST (RPE 14–16/20) Intensity and Volume: AT (CT) (20 min with/without rest), ST (EXP) 3times a week (3 sets, 30–60 sg) Rest: ST: 30–60 sg (between exercises), 60–120 sg (between sets) Supervised: yes Setting: Hospital	T1-T2: - ↑ Jumping height (∆12.4%) - ↓ TUG (∆-19.4%) ↑ STEO/↑ STEC sway path (∆5.5%, (∆7.7%))
(Pahl et al., 2020) [54]	RCT T1: Pre HSCT (Admission) T2: Post HSCT (D) T3: PostHSCT (180 days D)	- EXP: *n* = 18 (7 female), 55 years (50–63) - CT: *n* = 26 (7 female), 56 years (32–63)	ALL, AML, CLL, CML, Lymphoma, MDS, MF, MM, SAA, Septic granulomatosis,	Type: ST with vibration (EXP), mobilization of the spine and stretching (CT) Duration: N/R Intensity: N/R Volume: WBV/ST (EXP) 5times a week (20 min), mobilization and stretching (CT) 5times a week (20 min) Supervised: yes Setting: Hospital	T1-T3: - VO_2_: ↑ EXP/↓ CT (∆5.3%; (∆-11.8%) - P max: ↑ EXP(∆16.3%) - Knee flexors/extensors: ↓ CT (∆-23.1%; ∆-3.9%) - JH: ↓ CT (∆-3.3%) - BMI: ↓ EXP/↓ CT (∆-3.1%; (∆-12.4%)
(Almeida et al., 2020) [55]	RCT T1: Pre HSCT (Admission) T2: Post HSCT (D)	- EXP: *n* = 15 (7 female), 46.6 years (35.1–52) - CT: *n* = 16 (6 female), years 37.5 (39.5–53.6)	AML, Amyloidosis, HL, LHN, MM, SAA	Type: IMT (EXP) and AT (stationary bicycle), ADL, stretching, coordination, and balance (EXP/CT) Duration: N/R Intensity: IMT (40% MIP) Volume: IMT (EXP) 5 times a week (10–20 min, 12–16 diaphragmatic breathing per min), AT (EXP/CT) 5 times a week (10–20 min at 50–70% HR max), ADL weekends (EXP/CT), stretching (EXP) (CT) 5 times a week (10–20 min) Supervised: yes Setting: Hospital	T1-T2: - MIP: ↑ EXP (∆19.3%)

**Abbreviations**: A = admission; AA = aplastic anemia; ADL = activities of daily living¸ AML = acute myeloid leukemia; ALL = acute lymphoid leukemia; APL = acute promyelocytic leukemia; AT = aerobic training; BMI = body mass index; CLL = chronic lymphocytic leukemia; CML = chronic myeloid leukemia; CR = chair-rising test; CRF = cardiorespiratory fitness; CRT = chair-rising test; CT = control group; D = discharge; DCT = daily cardiovascular training; EXP = experimental group; EX fit = initially fit patients in the exercise group; EX unfit = initially unfit patients in the exercise group; Ext = extension; FRM = fat-free mass; Flex = flexion; H = hospitalization; Hb = hemoglobin; HL = Hodgkin’s lymphoma; HRmax = heart rate maximal; HSCT = hematopoietic stem cell transplantation; IMT = inspiratory muscle training; JH = jump height; LBW = lean body weight; LHN = non-Hodgkin’s lymphoma; KPS = Karnofsky Performance Status; MDS = myelodysplastic síndrome; MIP = maximal inspiratory pressure; MF = myelofibrosis; MM = múltiple mieloma; MPS = myeloproliferative síndrome; MW = Morbus Waldenström; PCMJ = power output during counter-movement jump; PID = primary immune deficiency; Plts = platelets; PMF = primary myelofibrosis; Pmax = maximum power output; PNH = paroxysmal nocturnal hemoglobinuria; RCT = randomized controlled trial; RPE = rate of perceived exertion; ST = strength training; SAA = severe aplastic anemia; ST_EC_ = semi-tandem stance with eyes closed; ST_EO_ = semi-tandem stance with eyes open; STEER = Strength Training to Enhance Early Recovery; TUG-3 m = timed up and go test (3 m); VO_2_= oxygen uptake; WBV = whole body vibration; WM = Waldenstrom macroglobulinemia; 2MWT = 2 min walk test; 6MWT = Six-minute walk test; 30CST = 30-s chair stand test. **Symbols:** ↔ = no improvement; ↑ = increase; ↓ = decrease; Min = minutes; n = sample size; N/R = not reported; Reps = repetitions; Sg = seconds; 1 RM = 1 repetition máximum; ^a^ (median ± SD); ^b^ (mean ± SD).

**Table 3:** Studies that have analyzed the effects of an exercise program after HSCT, with main results in physically related variables.

Study	Study Design	Sample Size by Group (Sex), Age (Mean ± SD; Range)	Primary Cancer	Intervention	Main Results in Physical Related Variables
(Shelton et al., 2009) [34]	RCT T1: Pre HSCT (A) T2:Post HSCT (4 weeks)	- Supervised: *n* = 26, (9 female), 43.65 ± 13.18 ^a^ years - Self-directed: *n* = 27, (11 female), 93 ± 11.66 ^a^ years	ALL, AML, CLL, CML, HD, Lymphoma, LHN	Type: AT (stationary bicycle, treadmill), ST (weight machines) Duration: 4 weeks Volume and intensity: AT: 3 days a week (20–30 min 60–75% HR max and BFI: 0–10), ST: 3 days a week EXP supervised (1–3 sets of 10 reps), EXP self-directed (1–3 sets of 10–15 reps). The AT and ST increased every third visit; if extreme fatigue, resistance was reduced to the previous level. Supervised: yes Setting: hospital/home	T1-T2: Supervised= - ↓ 50 FWT (∆-13.7%) Self-directed = - ↑ 6MWT (∆12.0%) - ↔ Immune system
(Barğı et al., 2016) [35]	RCT T1: Pre HSCT (A) T2: Post HSCT (6 weeks)	- EXP: *n* = 20, (8 female), 34.10 ± 12.65 ^a^ years - CT: *n* = 18, (6 female), 39.11 ± 12.57 ^a^ years	AA, ALL, AML, CML, Fanconi anemia, MDS, MM, LHN, PNH	Type: Respiratory muscle Duration: 6 weeks Volume and intensity: AT: 7 days at week (speed progressively increased at 1 min intervals walking at 12 stages/30 min rest between 2 tests with FIS (1–4). Diaphragmatic breaths: 7 days at week (EXP) (15 sg/25–30 breaths/5–10 resting IMT at 40% of MIP), (CT) (received sham IMT at fixed workload, 5% of baseline MIP with MMRC (0–4)) Supervised: yes Setting: hospital/home	T1-T2: - ↑ MISWT (∆8.0%) - ↑ 6MWT (∆5.8%) - ↑ MIP (∆36.6%) - MEP:↑ EXP/↑ CT (∆15.3%) - FEV1/FVC: ↓ CT (∆-1.1) - ↓ MMRC (∆-37.5%) - ↔ Immune system
(KIM & KIM, 2006) [41]	RCT T1: Pre HSCT (A) T2: Post HSCT (6 weeks)	- EXP: *n* = 18 (10 female), 32.9 ± 7.0 ^a^ years - CT: *n* = 17 (8 female), 34.3 ± 7.8 ^a^ years	AA, ALL, AML	Type: bed exercise intervention: Stretching, mobility, relaxation breathing Duration: 6 weeks Volume and intensity: Stretching and mobility bed exercise intervention: 7 days a week, 3 × 10 min: preliminary exercise. Relaxation breathing and finish exercise. Supervised: yes Setting: hospital	T1-T2: Lymphocytes: ↓ CT
(Persoon et al., 2017) [50]	RCT T1: Post HSCT (A) T2: Post HSCT (18 weeks)	- EXP: *n* = 54 (22 female), 53.5 years (20–67) - CT: *n* = 55 (18 female), 56 years (19–67)	HL, MM	Type: AT (stationary bicycle), ST (weight machines) Duration: 18 weeks Intensity and volume: AT 1–8 week twice a week (blocks of 30 sg at 65% MSEC alternated with blocks of 60 s at 30% MSEC), from 9–18 weeks (blocks of 30 s at 65% MSEC alternated with blocks of 30 sg at 30% MSEC), ST 1–12 weeks twice a week (2 sets/10 reps 60–80% 1 RM, from 13–18 weeks once a week (1 set/20 reps 35–40% 1RM). Supervised: Yes Setting: local physiotherapy	T1-T2: - No changes
(Bird et al., 2010) [51]	RCT T1: Post HSCT (A) T2: Post HSCT (6 months)	- EXP: *n* = 29 (13 female), 57 years (44–53.5) - CT: *n* = 29 (7 female), 52 years (42.5–63)	Leukaemia, Lymphoma, Myeloma	Type: AT, relaxation Duration: 10 weeks Intensity and volume: AT CEXP) 1–10 weeks (a series of circuit training exercise), relaxation (guided imagery). AT (CT) 1–10 weeks 3 times/week (home-based exercise program) Supervised: Yes Setting: Hospital/Home	T1-T2: - No changes
(Oechsle et al., 2014) [52]	RCT T1: Post HSCT (A) T2: Post HSCT (After intervention)	- EXP: *n* = 17 (7 female), years 51.7 ± 13.3 ^c^ - CT: *n* = 17 (7 female), years 52.9 ± 15.4 ^c^	AML, LHN, MM, Germ cell tumor	Type: AT (stationary bicycle), ST (elastic bands, bodyweight) Duration: 21 days Intensity and volume: AT 5 times a week (10–20 min), ST 5 times a week (20 min, 2 sets of 16–25 reps at 40–60% of 1 RM) Rest: AT (regular pauses until recuperated to 66.6%) Supervised: Yes Setting: Hospital	T1-T2: - CT: ↓ VO_2_ (∆-26.0%) - EXP: ↑ VO_2_, VE and strength upper limbs (∆11.3%; (∆21.8%; (∆35.7%)
(Hacker et al., 2011) [37]	RCT T1: Pre HSCT-Post-HSCT (After 8° day) T2: H-Post-HSCT (1–6 week)	- EXP: *n* = 9 - CT: *n* = 10 *n* = 19, (5 female) 46.26 years (16.23)^c^	AML, Lymphoma	*Type:* ST (elastic bands, bodyweight) *Duration:* 6 weeks *Volume and intensity:* ST 1–6 weeks 3 times a week (1–2 sets of 8–10 reps of RPE (13/20) *Supervised:* yes *Setting:* hospital/home	T2: - ↑ 30CST: EXP/CT (∆-12.7%; ∆-12.0%) - ↓ TNSU: EXP/CT (∆-18.9%; ∆-20.3%) - ↔ Immune system
(Knols et al., 2011) [43]	RCT T1: Pre HSCT (A)-Post HSCT (D) T2: Post HSCT (D)-Post HSCT (3 months)	- EXP: *n* = 64 (26 female), 46.7 ± 13.7 years (18–75) ^a^ - CT: *n* = 67 (28 female), 46.6 ± 12 years (20–67) ^a^	ALL, AML, Amyloidosis, CLL, HL, LHN, Lymphoma, MM, osteomyelofibrosis, testicular cancer	*Type:* AT (stationary bicycle), ST (dumbbell) *Frequency:* 2 days/week *Duration:* 12 weeks *Intensity and volume:* AT twice a time (50–70% to 80% HRmax) *Supervised:* yes *Setting*: fitness center/physiotherapy practise	T1-T2: - Knee extensión: ↑ EXP/↑ CT (∆26.2%) - Walk speed: ↓ EXP/↓ CT (∆-11.9%; ∆-2.4%) - 6MWT: ↑ EXP/↑ CT (∆17.7%; ∆9.8%) - ↔ Immune system
(Schumacher et al., 2018) [44]	RCT T1: Pre HSCT (A)-Post HSCT (14 days) T2: Pre HSCT (A)-Post HSCT (30 days)	- EXP: *n* = 19 (3 female), 56 years (21–65) ^b^ - CT: *n* = 23 (14 female), 56.5 years (23–69) ^b^	AML, CLL, CML, LHN, MDS, MM, Teratoma	*Type:* AT (walking, step), ST (elastic bands, bodyweight), *Stretching, Wii sports, Wii fit program, Wii balance* *Duration:*100 days *Intensity:* N/R *Frequency: 5 days/week* *Supervised:* yes *Setting:* hospital	T1-T2: - 2MWT: ↓ EXP/↓ CT (∆-1.7%; ∆-4.9%) - Treadmill: ↓ EXP/↓ CT (∆-13.2%; ∆-3.8%) - L HGS: ↓ EXP/↓ CT (∆-9.3%; ∆-8.1%) - ↔ Immune system
(Van Dongen et al., 2019) [53]	Multicenter RCT T1: Post HSCT (Baseline) T2: Post HSCT (After exersice or similar time point in the CT) T3: Post HSCT (12 months later)	- EXP: *n* = 54 (22 female), 52 ± 11 ^c^ years - CT: *n* = 55 (2 female), 53 ± 12 ^c^ years	HL, MM	Type: AT (stationary bicycle), ST (weight machines) Duration: 18 weeks Intensity and volume: AT 1–8 weeks twice a week (2 × 8 min, alternating 30 sg at 65% and 60 sg at 30% MSEC), 9–12 weeks twice a week (2 × 8 min, alternating 30 sg at 65% and 30 sg at 30% MSEC), 13–18 weeks once a week (2 × 8 min, alternating 30 s at 65% and 30 sg at 30% MSEC), ST: 1–12 weeks twice a week (2 sets of 10 reps at 65–80% of 1-RM), 13–18 weeks once a week (2 sets of 20 reps at 35–40% of 1-RM) Supervised: Yes Setting: Hospital	T1-T3: - No changes

**Abbreviations:** A = admission; AA = aplastic anemia; AML = acute myeloid leukemia; ALL = acute lymphoid leukemia; AT = aerobic training; CLL = chronic lymphocytic leukemia; CML = chronic myeloid leukemia; CRF = cardiorespiratory fitness; CT = control group; D = discharge; EXP = experimental group; FEV1/FVC = forced expiratory volume in the first second/forced vital capacity; FIS = fatigue impact scale; HD = Hodgkin’s disease; HGS = hand grip strength; HL = Hodgkin’s lymphoma; HSCT = hematopoietic stem cell transplantation; LHN = non-Hodgkin’s lymphoma; MDS = myelodysplastic síndrome; MEP = maximal expiratory pressure; MIP = maximal inspiratory pressure; MISWT = modified incremental shuttle walking test; MM = múltiple mieloma; MMRC = Modified Medical Research Council; MSEC = the maximal short exercise capacity; PASE = Physical Activity Scale for the Elderly; PNH = paroxysmal nocturnal hemoglobinuria; RCT = randomized controlled trial; ST = strength training; SWT = shuttle walk test; TNSU = time needed to stand up; VO_2_= oxygen uptake; 6MWT = six-minute walk test; 30 CST = 30-s chair stand test; 50 FWT = 50-foot walk test. **Symbols:** ↔ = no improvement; ↑= increase; ↓ = decrease; Min = minutes; n = sample size; N/R = not reported; Reps = repetitions; Sg = seconds;1 RM = 1 repetition máximum; ^a^ (median ± SD; range); ^b^ (median ± SD); ^c^ (mean ± SD).

## Data Availability

Not applicable.
